# Relaxation for Dentists

**Published:** 1883-02

**Authors:** 


Relaxation for Dentists.—Of all the plain, though sometimes unpalatable, truths which were told Americans by that distinguished scientist, Herbert Spencer, during his late visit to this country, there were none which should sink deeper into our hearts than the charge that we are too utterly devoted to business, and do not take sufficient time for relaxation. The average American has a highly-wrought, nervous organization, partially due to a peculiar climate, but constantly exaggerated by the incessant whirl and bustle of excitement in which we delight, and by the intense and feverish application to business so characteristic of us as a people. Even the very few pleasures which we allow ourselves are tainted by this over-exertion, and our hours of ease are made laborious by a constant state of unrest. If the typical American travels, though it be solely for recreation and pleasure, it must be by express train. If he attend the theatre or opera, or goes to a concert, he does not arrive until after the commencement, while he leaves just before the close, and all the intervals are filled up with his newspaper or note-book. There is no quiet, leisurely repose, no shaking off of worldly cares, no forgetting the shop, no utter abandonment to entire leisure. Because of this, we are too often the prey of certain ailments to which the average European is almost a stranger. Whatever illness attacks the American is almost sure to be complicated with some nervous disorder. The Englishman eats an amount of hearty food which astounds an American. According to our dietary laws, he should suffer all the agonies of dyspepsia, whereas, that disease is almost a stranger to him. His digestion is assisted by an amount of vigorous out-of-door exercise, and by hours of relaxation and rest to which the American is a stranger. The fondness of the Englishman for wading up to his knees in ice-cold water that he may secure a few worthless fish, or for tramping all day through mud and mire for the purpose of shooting a few birds which he is not allowed to carry off the premises, is to us inexplicable. But the truth is, John Bull is a fine, sturdy animal, and he believes in cultivating and gratifying the animal part of his nature. The American is too often so utterly engrossed in the accomplishment of some desired purpose, that he neglects the physical part of his being, upon which his mental powers are so largely dependent for healthy action, and, in consequence, breaks down with his set task, perhaps, but half accomplished.

It is high time for the dental profession to take a new departure in this respect. There are few avocations which make greater demands upon the vital forces than does ours. The mind of the dentist must be constantly alert to meet the ever-varying complications which arise. No two operations are performed exactly alike, and it requires the strictest attention to details, that imperfections may not creep in. A moment's inadvertence may ruin his finest work.

Then again, he is always laboring upon sensitive tissue, often for timid, shrinking, apprehensive patients, and thus his sympathies are constantly excited, and his nerve tension extreme. Add to this the fact that his occupation requires him to often remain in a constrained, unnatural position, that his life is an inactive one, within doors, too often in an overheated room, that he is subjected to the exhalations of diseased people, with offensive, fetid breaths, and there is little wonder that in middle life the operative dentist usually begins to experience a premature old age.

All these adverse conditions might be successfully combated by a systematic course of outdoor exercise, but the busy operator needs every hour of sunlight for the performance of his task, which can only be accomplished in a strong light. The very hours which are best fitted for recreation, the light of the blessed sun which should regularly fall upon him during relaxation, must be utilized in the service of others, and he lives in a forcing house which, while it may tend to develop certain faculties, is prejudicial to perfect physical health.

Dental practice in this country is far different from that of Europe. There are many more long, tedious, and difficult operations performed here. We see fewer patients in a day, and spend more time over each one. Then, too, ours is usually a more *personal* practice than is that of our professional brethren in Europe. Patients demand the exclusive attention of the dentist, and are not content to submit themselves to an assistant for minor operations. A practice here, while it is more readily obtained, is also more quickly lost. People are less tenacious of old connections, and seek a new dentist whenever they are unable to command the personal services of the old one, so that to retain a practice once gained, it is requisite that the operator have few holidays. Taking little relaxation themselves, our people are not tolerant of those who, professing to be at their service, are not eternally at their beck and call. There may be a few favored practitioners who can afford to ignore these unreasonable demands, but the average dentist must comply or starve.

We have no thorough system of pupilage, nor have we the class of trained assistants that are easily obtained in Europe. Here, any man is as good as his master, if not a little better, and as soon as a pupil or assistant has acquired sufficient skill to extract a tooth and insert a cheap rubber substitute, he straightway strikes out for himself, and appeals for support to such patients of his preceptor as he has been permitted to meet, urging that he has been enabled to acquire all the methods and knowledge of the latter, and promising the same results for half the price. In Europe, it is not thus easy to step from a subordinate to an independent position. Business moves in certain well-defined grooves, and public sentiment is not thus tolerant of one who desires to supplant an old-established practitioner. The student or assistant must enter upon practice with the good will of his preceptor, if it be in his immediate neighborhood.

But it is not alone the public sentiment of the older countries which condemns such acts. The law takes cognizance of the rights of the master, and forces the student to pass a sufficient term of pupilage to become thoroughly schooled in his business, before he begins an independent career. It is not sufficient for him to learn how to prepare a vulcanite denture. He must be versed in metallurgy and prepared to work gold. In consequence of all these things, there is, in Great Britain and upon the Continent, a class of assistants or journeyman dentists who are content to remain subordinates throughout their lives. They are quite competent to do all the mechanical work without close supervision, and to perform the minor operations of the surgery. They can take the place of the dentist while he may be enjoying relaxation, and patients do not manifest the annoyance at being served by them which is common in this country, where every man is so exceedingly jealous of his independence, and so apprehensive lest his dignity be compromised, that he declines to be served by a subordinate and demands the presence of the principal.

Our methods of conducting a dental practice have their compensating advantages, but they chiefly accrue to the patient. We are quick to seize upon any idea which will result in our good, but the whole fabric of our social institutions is so widely different from that of the old country that it is difficult to see wherein lies the remedy for the constrained overwork of our best dentists, unless it be through the slow growth of an enlightened public opinion. The safeguards which, in England, for instance, are sufficient to protect a dentist from spoliation of his practice through the introduction of a competent assistant to his patients are inadequate here. If he bind such an one under heavy penalty not to engage in independent practice in his neighborhood, the chances are that the courts would not enforce the penalty, and even should they do so, public opinion would condemn the practitioner who would thus endeavor to restrain unfair competition, and the notoriety of the case would work only evil to the senior, while it would profitably advertise the junior. Recognizing these facts, many of the better class of dentists refuse to receive pupils, decline to employ competent assistants, and are compelled to get along as best they may, with the help of a girl to clean their instruments and keep everything in order. Dental legislation is doing much to better this condition, mainly through the production of a better educated public opinion. The remainder must rest with the dentists themselves. If, in every city or community, all should agree upon taking one day, or even half a day in each week for needful recreation, no one would be injured, while all would be materially benefited. It is universally admitted that the rest of the Sabbath is profitable, not alone upon moral grounds, but as well from sanitary considerations. The man who works six days in the week and rests upon the seventh, will, in the long run, do more and better work than he who gives himself no rest. But public sentiment demands that, while Sunday shall be given up to rest, it shall not be devoted to recreation, and the dentist who proposes to secure the respect of church-going people must take another day for the out-door exercise so necessary for his physical well being. Although teachers labor but about six hours each day, public opinion demands that the schools shall be closed on Saturdays either for a whole or half holiday. The lawyer, although not confined to such close office hours as the dentist, is seldom found in on Saturdays. Clergymen almost invariably reserve Mondays to themselves. Physicians keep but few office hours, and the rest of their time is spent out of doors, either in visiting patients or taking needful exercise. A system of early closing on Saturdays is fast becoming an established custom in shops and stores and manufactories, that proprietors and employees may have time for personal enjoyment. An old and venerated institution teaches us that wise men divide their time into three parts, whereby they obtain eight hours for the service of God and a distressed worthy brother, eight for their usual avocations, and eight for refreshment and sleep. The hard-worked dentist cannot cease from his labors for one-third of his waking hours. Why should it not be possible for the profession generally to agree upon a regular half holiday for Saturday?

The time, too, must eventually come when we shall have among us a class of competent assistants, and that without prejudice to one's practice. To this end every dentist should labor, by encouraging a public sentiment which will sustain a dentist in demanding and enforcing sufficient guarantees, that the privilege of introduction and recommendation to one's patients shall not be abused by the trusted junior.

In this country all needful reforms must be accomplished by concerted action and through a popular vote. So imperative do we believe to be this need for more recreation for dentists that we desire earnestly to commend the subject to the consideration of dental societies, that some remedy for the evils under which the profession is laboring may be devised. In the larger cities it ought not to be difficult for the operative dentists to agree upon a day, or half a day, to be strictly observed as a holiday, when offices would be closed, or left in charge of some one empowered to make appointments.

Many of the States have dental laws which forbid the commencement of an independent practice by any one who has not the requisite qualifications. While it may be difficult, nay, impracticable in many cases, to enforce such enactments, yet we believe they will result in good, through a wholesome enlightenment of the public mind, and thus, in the end, bring about a proper settlement of the vexed question of dental pupilage, with the final result of securing a class of trained assistants who will lighten the labors of an overworked profession.

